# Vision-Based People Counting and Tracking for Urban Environments

**DOI:** 10.3390/jimaging12010027

**Published:** 2026-01-05

**Authors:** Daniyar Nurseitov, Kairat Bostanbekov, Nazgul Toiganbayeva, Aidana Zhalgas, Didar Yedilkhan, Beibut Amirgaliyev

**Affiliations:** 1 KazMunayGas Engineering LLP, Astana 010000, Kazakhstan; 2 Al-Farabi Kazakh National University, Almaty 050040, Kazakhstan; 3 Research and Innovation Center “Smart City”, Astana IT University, Astana 010000, Kazakhstan

**Keywords:** people counting, computer vision, multi-sensor fusion, depth camera, object tracking, real-time processing, public transport monitoring, smart mobility

## Abstract

Population growth and expansion of urban areas increase the need for the introduction of intelligent passenger traffic monitoring systems. Accurate estimation of the number of passengers is an important condition for improving the efficiency, safety and quality of transport services. This paper proposes an approach to the automatic detection and counting of people using computer vision and deep learning methods. While YOLOv8 and DeepSORT have been widely explored individually, our contribution lies in a task-specific modification of the DeepSORT tracking pipeline, optimized for dense passenger environments, strong occlusions, and dynamic lighting, as well as in a unified architecture that integrates detection, tracking, and automatic event-log generation. Our new proprietary dataset of 4047 images and 8918 labeled objects has achieved 92% detection accuracy and 85% counting accuracy, which confirms the effectiveness of the solution. Compared to Mask R-CNN and DETR, the YOLOv8 model demonstrates an optimal balance between speed, accuracy, and computational efficiency. The results confirm that computer vision can become an efficient and scalable replacement for traditional sensory passenger counting systems. The developed architecture (YOLO + Tracking) combines recognition, tracking and counting of people into a single system that automatically generates annotated video streams and event logs. In the future, it is planned to expand the dataset, introduce support for multicamera integration, and adapt the model for embedded devices to improve the accuracy and energy efficiency of the solution in real-world conditions.

## 1. Introduction

With the rapid growth of urban areas and the increasing demand for efficient public transport, accurate assessment of passenger traffic is becoming a key element in the planning and management of transport systems. Understanding how many people use buses, trams, and the subway at different hours of the day allows transport agencies to optimize schedules, rationalize resources, and improve passenger comfort. According to UN forecasts [1], by 2050, more than 68% of the world’s population will live in cities, which makes automated monitoring of passenger traffic an important component of the smart cities concept. However, current vision-based passenger counting systems still struggle with high-density environments, occlusion, and unstable lighting conditions. This study addresses the research question: how can detection and tracking algorithms be optimized to ensure accurate passenger counting under real-world transport conditions?

In the last few years, the development of technologies such as computer vision and deep learning has introduced new applications of automated people counting in public transport areas. Camera types like RedGreenBlue (RGB), depth, and multisensor are the main sources of these technologies which working together can identify, monitor, and count passengers with very high precision. Different research projects have confirmed the effectiveness of convolutional neural networks (CNNs) and object detection models like YOLO, RetinaNet, and Faster region-based convolutional neural network (R-CNN) for these applications. The practice of moving into the real world is still beset with issues such as occlusions, different light conditions and privacy matters (for instance, ensuring compliance with General Data Protection Regulation (GDPR)).

This paper reports the development and evaluation of the collection and processing of data for detecting people in urban transport systems. The study examines various methodologies, datasets, and computational approaches. Particular attention is paid to existing detection systems, their performance metrics, and practical difficulties in implementation. The aim of this research is to develop and validate an integrated detection–tracking architecture that achieves robust counting accuracy in crowded and dynamic public transport scenarios. The main contributions of this work include: (1) a task-specific modification of the DeepSORT tracker, (2) a new dataset collected in real public transport settings, and (3) a unified pipeline that performs detection, tracking, and automatic event-log generation.

## 2. Related Work

To determine the place of this research in the existing scientific context, the section is devoted to a critical analysis of modern methods of automatic passenger detection and counting. The analysis is built on the most significant tasks that were already solved in earlier research and remain relevant for further advancement of the field.

In recent years, experts have focused on the use of computer vision and deep learning technologies to create more accurate and reliable passenger counting systems in public transport. The main issues for researchers are object overlap, lighting variations, and maintaining identity when people change positions. The following sub-sections will present the most important methods that have been proposed in the scientific literature and will also include their analytical evaluation regarding effectiveness and limitations.

### 2.1. Handling Occlusions and Crowded Scenes

One of the key limitations of human counting systems is their dependence on the degree of overlap of objects and crowd density. One of the studies of Ren et al. [2] presented an improved modification of the YOLO architecture called YOLO-PC. It used a 9 × 9 grid, which provides a more accurate definition of the boundaries of objects and an improved counting mechanism. This approach allows not only to improve the recognition accuracy, but also the frame rate > 40 FPS (Frames Per Second). However, with a large number of people, when objects partially overlap each other, there can be seen that the effectiveness of the model has noticeably decreased. Similarly, Baumann et al. [3] proposed a two-step approach that combines the RetinaNet detector with a recurrent neural network to account for time dependencies between frames. Despite the fact that the system showed a high accuracy (mAP of 97.3%), it required significant computing resources, and could not always work in real time with high stage load. These examples show that when working in dynamic conditions with overlaps, one has to find a balance between accuracy and stability of the model.

The study by Terven et al. [4] examines in detail the YOLOv8 architecture, its learning principles, and improvements compared to previous versions. In paper of Li et al. [5], lightweight YOLOv5 variants using inner cross bottleneck (ICB) and outer cross bottleneck (OCB) modules were proposed, which reduced the network size by 31% and achieved processing speeds of up to 40 FPS without loss of accuracy. Lin et al. [6] presented solutions based on CNNs that combine the tasks of detecting, counting, and recognizing passenger actions recorded by ceiling-mounted cameras, utilizing double error correction algorithms to increase reliability. The effectiveness of instance segmentation methods was also investigated, especially in the YOLOv5-Seg and YOLOv8 models. It was noted that the use of masks significantly improves the accuracy of object localization, especially in complex scenes with overlaps, but at the same time, such models require significant computing resources, which limits their use in systems where high real-time processing speed is critically important.

Xu et al. [7] proposed the ESE-Seg framework for fast instance segmentation based on explicit encoding of the object shape using radial representation and Chebyshev polynomials. This approach allows you to create accurate masks with computational complexity comparable to object detection methods. In addition, the researchers examined the use of models designed to analyze human postures, such as OpenPose and HRNet, which provide effective determination of body position and orientation. Such solutions are useful in conditions of high passenger density, but the disadvantage is that they remain sensitive to the camera’s viewing angle and require significant computing resources.

In their work, Yamazaki et al. [8] presented a compact architecture for assessing posture, specifically designed for devices with limited computing capabilities. Their proposed model includes a lightweight encoder-decoder with simplified deconvolution layers and uses the methods of distillation and quantization of models. According to the experimental results, the proposed system achieved 94.5% accuracy compared to HRNet on the Common Objects in Context (COCO) dataset, while reducing computing costs by up to 3.8% and providing speeds of up to 60 FPS on the NVIDIA Jetson AGX Xavier device. A modification of YOLO that combines bounding boxes and key points (YOLO-Pose) was also considered. This model demonstrated balanced performance in terms of accuracy, speed, and robustness, making it promising for real-world transportation systems. Chen et al. [9] further improved YOLO-Pose for pose estimation in dense crowds by incorporating a CBAM attention module and replacing the CIOU loss function with EIOU. Their model provides more accurate keypoint localization and outperforms YOLOv8 under occlusion conditions.

### 2.2. Dealing with Lighting Variability and Sensor Limitations

Changes in illumination and the features of the sensors used significantly affect the accuracy of passenger detection systems in public transport. In the study of Sun et al. [10], RGB-D cameras and depth-based feature extraction methods were used to solve this problem, which made it possible to reliably determine the position of a person’s head by reconstructing a three-dimensional point cloud. The accuracy achieved 92%, but the implementation depended on special equipment (for example, Kinect V1) and required precise camera calibration, which made large-scale implementation difficult. In the work of Kusuma et al. [11], the YOLOv4 architecture was used, trained by the knowledge transfer method on a mixed dataset including images from open sources and proprietary samples. Despite the rather high values (mAP of 72.68%), the accuracy was noticeably reduced in low light or glare. To increase the resistance to various light conditions, it was necessary to carry out large-scale data augmentation. In general, research shows that ensuring the stable operation of algorithms with changes in illumination remains a serious challenge, especially for systems that use only RGB cameras and are installed directly on vehicles.

### 2.3. Re-Identification and Tracking-Oriented Approaches

Preserving the identity of passengers on consecutive frames and when using multiple cameras is crucial for accurate counting. Classical tracking algorithms such as SORT [12] and DeepSORT [13], which link detected objects over time, are widely used for this task. Later, more advanced solutions appeared, such as FairMOT [14], which combines the tasks of detection and re-identification into a single multimodal architecture. This approach significantly improved the accuracy of multi-object tracking (MOTA ≈0.67, IDF1 ≈0.80). However, such models require significant computing resources and are sensitive to camera movements and changes in human postures. Other developments, such as ByteTrack [15], improved stability by accounting for low-confidence detections, while BoT-SORT [16] improved identification quality by improving DeepSORT results. A cross-chamber approach based on CNNs was also proposed by Lin et al. [6], combining the tasks of detecting, counting, and recognizing actions in order to prevent double counting. However, such systems have been tested only in laboratory conditions, which limits their practical application.

Thus, creating reliable and lightweight tracking solutions that can work on embedded and peripheral devices remains an urgent task. In addition, it can be noted that the quality and diversity of datasets play an important role in increasing the stability and generalizing ability of models. In recent years, more and more research has focused on the development of specialized datasets and the integration of data from various sensors, which is discussed in the next section.

### 2.4. Dataset Development and Multisensor Fusion

The lack of large, diverse, and labeled datasets remains a bottleneck in this area of research. In one of the papers of Sun et al. [10], a set of People Counting Dataset (PCDS) was presented, designed to count people from deep images, which significantly increased the reproducibility of experiments in this field. Radovan et al. [17] have reviewed approaches to multisensory integration, including combining visual, infrared, and Wi-Fi/RFID data. Such solutions have shown high resistance to overlaps and changing environmental conditions, but require expensive equipment, complex calibration, and raise privacy concerns.

Recent works of McCarthy et al. [18] and Konrad et al. [19]) have examined video methods for assessing vehicle occupancy using wide-angle and external cameras. These approaches provided high accuracy, but had limited scalability due to sensor placement features. All these results highlight the continuing shortage of standardized and publicly available RGB datasets for public transport, especially in conditions of uncontrolled lighting and traffic.

For a more visual representation of the main technical areas and their characteristics, Table 1 provides a comparison of recent studies on passenger detection and counting. It reflects the differences in the datasets used, accuracy and processing speed, and shows the balance between high accuracy and real-time performance in different architectures.

As shown in Table 1, the highest accuracy can usually be achieved using complex architectures that require significant computing resources. This, in turn, limits the possibility of using them on embedded and peripheral devices. The comparative analysis highlights that the issues of sustainability, generalizing ability, and adaptation of models to real-world operating conditions remain unresolved. These issues are discussed in more detail in the next section on the research gaps and the proposed approach.

### 2.5. Research Gaps and Proposed Approach

Despite significant progress, a number of gaps remain unresolved:

- Limited robustness under dense crowding and occlusions, where detection overlap causes undercounting or double-counting.
- Sensitivity to illumination changes and varying camera orientations typical of onboard transport cameras.
- Insufficiently diverse datasets representing real-world public transport conditions.
- The lack of a single effective framework that integrates detection, tracking, and counting processes into an optimized pipeline suitable for deployment on peripheral devices.

The identified limitations became the basis for the development of the integrated YOLOv8–DeepSORT framework, which combines the tasks of detecting, tracking and counting passengers into a single video processing system. The proposed architecture includes object detection, segmentation, and multi-object tracking in a single computing pipeline. The system demonstrates stability in partial closures, lighting changes, and complex passenger movements, while maintaining the ability to work in real time even on equipment with limited computing resources. The framework is based on a specially created dataset of 4047 annotated images, which contributes to a better generalization of the model and opens up prospects for further developments, such as the integration of multiple cameras and the implementation of solutions for peripheral systems in real time. Dataset considers the images with high occlusion, variable lighting, diverse camera modalities, many group and crowd scenes.

Based on the identified research gaps and an analysis of existing approaches, the proposed methodology and implementation of the model are discussed in detail in the next section.

## 3. Methodology

### 3.1. Architecture

This paper presents a comparative overview of contemporary approaches to automatic passenger counting using computer vision technologies. The analysis focuses on three main aspects: object detection, instance segmentation, and human pose estimation, each offering distinct strengths and weaknesses depending on the application context.

To solve the problems of object detection, the YOLOv8 model [20,21,22] with pre-trained weights on the COCO dataset was used. The YOLOv8 architecture (You Only Look Once, version 8) is a modern real-time model specifically designed for computer vision tasks. The analysis also included the architectures of Faster R-CNN, YOLO, including versions YOLOv5 and YOLOv8, and RetinaNet. These models use bounding boxes to localize objects, combining high processing speed with an acceptable level of accuracy. However, with a high density of passenger traffic and partial closure of facilities, their efficiency decreases. To solve these problems, instance segmentation methods are used, which make it possible to more accurately determine the contours of objects using masks, which significantly improves the quality of detection. New versions of YOLO already integrate this functionality, making the models more versatile and effective when analyzing scenes with a high density of people.

We introduce a YOLO-based architecture for video-based people counting. Frames are processed to detect persons, associate persistent IDs, and update per-ID state histories relative to virtual lines defining a quadrilateral ROI. The counting of inputs and outputs (“IN/OUT”) is performed only after the complete intersection of this area, and the results are recorded and visualized on an annotated video.

**Figure 1:**
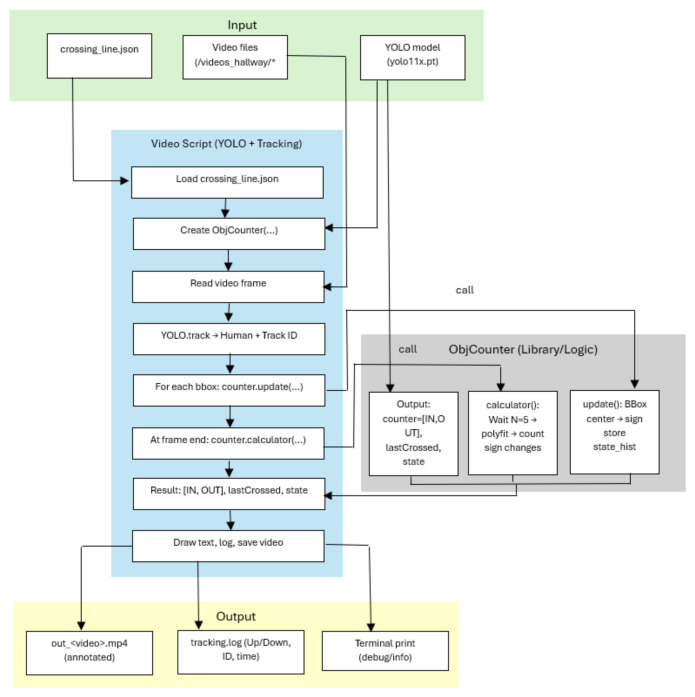
Video-Based People Counting: End-to-End Pipeline with YOLO, Multi-Object Tracking, and ObjCounter.

Figure 1 represents or analyzing human movements and evaluating bidirectional flow (entries and exits) in video sequences. It is implemented by integrating a YOLO-based object detection model and tracking algorithms. The system accepts structured input data such as video streams, YOLO model weights, and line intersection parameters, and generates annotated video files, structured event logs, and diagnostic information in the terminal.

### 3.2. Pre-Processing

The collection and formation of a dataset for the tasks of detecting people in transport and premises is an important stage in the development of computer vision systems, including passenger detection and counting modules. At the stage of preparing the dataset, about 2000 images were processed, pre-selected using object detection models. This approach made it possible to eliminate irrelevant frames and improve the quality of the source material for subsequent markup. The data was obtained from video recordings made by surveillance cameras installed in urban transport. This set is characterized by a high variability of shooting conditions, that is, differences in lighting, viewing angles, and passenger density, which makes it especially valuable for training computer vision models. A specialized platform was used for annotation [23], which ensures high accuracy and efficiency of the markup process. Each image underwent identification and marking of objects belonging to the “Person” class. The resulting annotations became the basis for training and further optimization of models aimed at solving the problems of detecting and counting passengers in real-world operation of transport systems.

**Figure 2:**
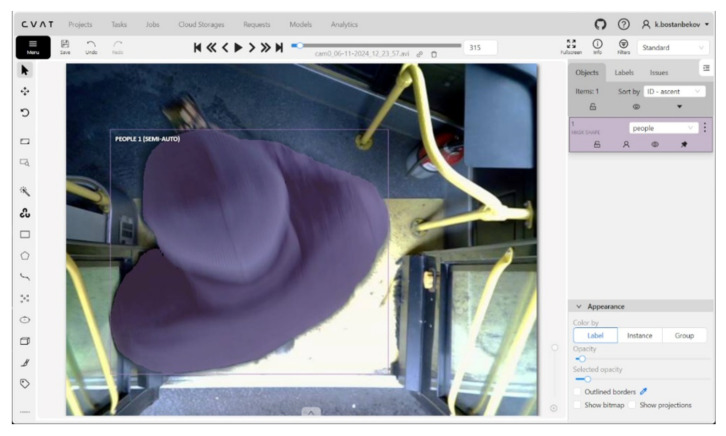
Example of object annotation for the “Person” class using mask segmentation in CVAT.

Figure 2 shows the annotation interface window in the CVAT (Computer Vision Annotation Tool) system, illustrating the process of polygonal segmentation of objects in a video surveillance scene. The image was captured by a camera installed in the entrance area of a city bus. Detected objects (passengers) are annotated either manually or semi-automatically using mask shapes, highlighted in purple. On the right side of the interface, lists of annotated objects with assigned IDs and class labels (“Person”) are displayed. The provided dataframe in Table 2 represents the annotated metadata of objects in the images, formatted in a structure similar to COCO. This data can be used for computer vision tasks such as detection, segmentation, or object tracking.

At the stage of dataset formation, the frames extracted from the videos were purposefully selected. The final sample included only those images in which the facial features of the passengers were not sufficiently distinguishable. This approach avoided the additional anonymization procedure and fully complied with the requirements of ethical data processing. The selected materials have been prepared and adapted for use in experiments related to deep learning tasks.

To train and test the models, a dataset structure was formed, consisting of twenty folders with video frames obtained from cameras installed inside city buses. In total, the sample included 4047 images containing 8918 annotated objects of the “Person” class. Of these, 16 folders (3293 images, 7322 objects) were used for training, and 4 folders (754 images, 1596 objects) for testing. The ratio of the training and test parts was 81% and 19% in terms of the number of images, and 82% and 18% in terms of the number of annotations. This distribution allowed us to maintain a balance between the representativeness of the test sample and a sufficient amount of data to train the model.

Additionally, some of the video materials were collected in the university corridor using depth and stereo cameras connected to the NVIDIA Jetson Nano computing module. To transfer streaming video to a storage server, a client-server architecture was implemented to ensure stable transmission and protection of video streams in real time.

**Figure 3:**
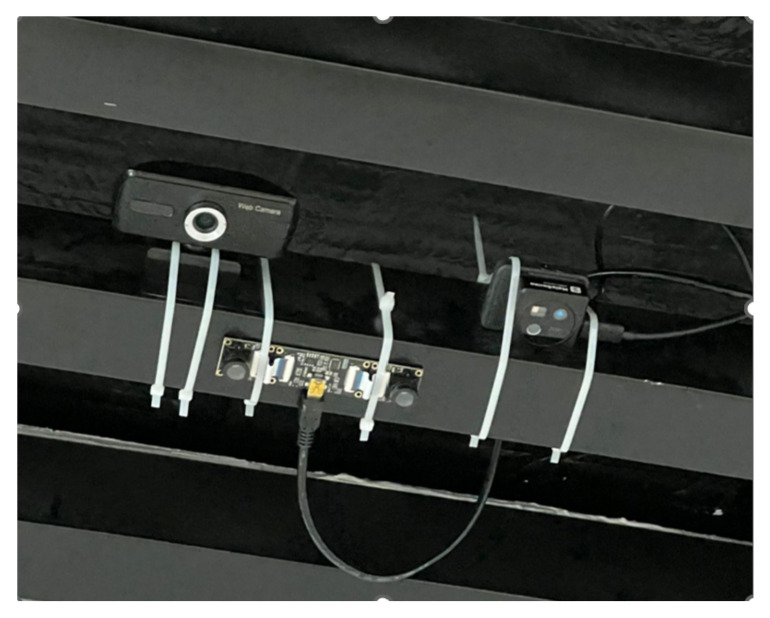
Three types of cameras were installed for dataset collection: RGB, stereo, and IR with depth.

To increase the stability of the algorithms, sensors with support for deep information (IR, stereo, RGB-D) were used, which made it possible to compensate for overlapping objects and background changes. Each camera type contributes exclusive advantages: RGB [24,25] is primary modality for training YOLOv8; provides high-resolution texture and color information. Stereo enables disparity-based depth estimation; useful for cases with occlusion and densely packed passengers, IR/Depth (MaixSense) provides reliable geometry in low illumination and supports volumetric (3D) scene understanding. Using three modalities helped capture day/night variation, occlusions, variable crowd density, and complex environment geometry, improving the generalization of the RGB-trained model. The following section, Figure 3, provides a list of cameras used and a diagram of the data collection process.

- **MaixSense A075V**—a compact intelligent AI camera with depth sensing capability, based on *active stereoscopy* technology.
- **3D Stereo VR USB Camera**—a passive stereo system consisting of two identical CMOS cameras integrated into a single housing with an adjustable inter-camera baseline.

During the data collection process at the university, the video was recorded using the NVIDIA Jetson Nano module, to which RGB-D and stereo cameras were connected. The shooting was carried out daily from 7:00 to 21:00, covering a wide range of lighting conditions and different types of people’s movements. All data was automatically stored on a central server for further processing and annotation.

**Figure 4:**
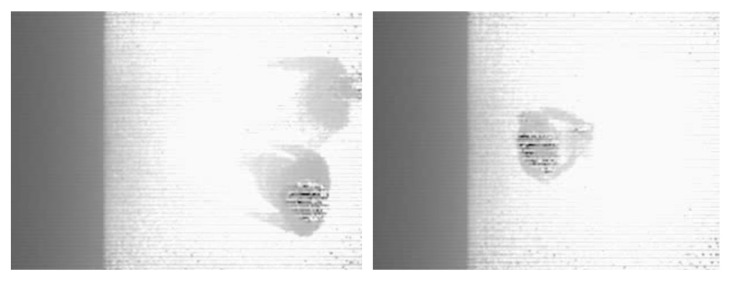
Depth map generation using the MaixSense A075V camera.

Figure 4 shows sample images taken from a MaixSense A075V stereo camera equipped with a depth mapping feature. The camera has an adjustable camera spacing and a fixed viewing angle, which allows to accurately capture the spatial position of objects. RGB, IR, and Depth video streams were recorded from the MaixSense A075V camera at 10-min intervals from August 25 to September 18, daily from 7:00 to 21:00. As a result, 5862 files were collected, and the total amount of data was 28.2 GB. Depth frames obtained from the MaixSense A075V camera are incorporated into the preprocessing pipeline for occlusion handling and spatial separation of passengers. Each depth map is synchronized with its corresponding RGB frame and uploaded to the annotation server, where depth values are used to refine object boundaries and assist in distinguishing closely spaced individuals in crowded scenes.

**Figure 5:**
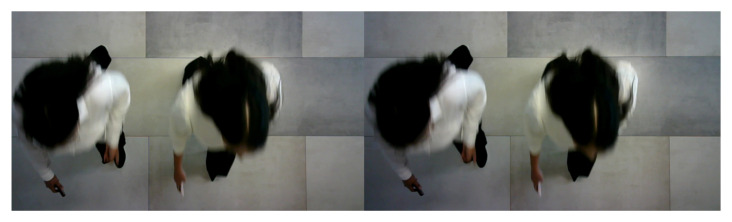
Images from the 3D Stereo VR USB camera.

Figure 5 shows an image obtained from a 720p 3D Stereo VR USB camera that provides synchronous recording of pairs of images from two sensors for subsequent depth assessment. To form a representative dataset, video streams were recorded at 10-min intervals from 7:00 a.m. to 9:00 p.m. during two collection stages: from August 27 to September 2 and from September 15 to September 18. In total, 712 videos were collected with a total volume of about 226 GB. The data obtained allows for a comprehensive analysis of passenger flows under various lighting conditions and scene density. The 3D Stereo VR USB camera produces two synchronized frames (Left and Right), which are stored as paired images for disparity estimation. These stereo pairs are processed on the server to generate disparity and depth cues, supporting the differentiation of overlapping passengers and improving scene understanding under dense or low-light conditions.

The collected dataset has become a reliable basis for the development and evaluation of computer vision algorithms focused on accurate passenger counting in dynamic urban environments. Combining high-resolution images with deep and stereo information provides a sufficient variety of data necessary for training neural networks capable of demonstrating stable results and good generalizing ability in real-world operating conditions.

The following section, Training the Neural Network Using Open and Proprietary Data, describes the learning process of the proposed model, which used both open and proprietary datasets. This integrated approach increases the accuracy, stability, and adaptability of the model, ensuring its reliable operation under various lighting conditions, scene densities, and camera configurations.

While the RGB dataset served as the basis for training the detection model, an additional algorithm based on deep information was developed to improve spatial understanding of the scene and support three-dimensional analysis.

### 3.3. Depth-Based Object Detection and Volume Estimation Algorithm

To analyze the video streams received from the depth camera, an algorithm was developed to detect changes and calculate the volumetric characteristics of objects based on depth data. The main idea of the method is to compare the current depth frame ($D_{bg}$) with a reference background image ($D_{bk}$). The depth difference is calculated as follows:

$$\Delta D=D_{bg}-D_{bk}$$

After threshold filtering and morphological operations, a binary mask of the regions of changes is formed. Each segmented region is analyzed using the connected component method, enabling the determination of object contours and the calculation of corresponding bounding boxes.

For each region based on depth data and internal camera calibration parameters ($f_{x}$, $f_{y}$, $c_{x}$, $c_{y}$) the area and volume of the object are calculated.

The transformation of pixel coordinates ($x_{p}$, $y_{p}$) and depth *z* into spatial coordinates (X,Y,Z) in the *camera coordinate system* is defined as:

$$\left\{\begin{array}{cc} X & =\frac{(x_{p}-c_{x})\;Z}{f_{x}},\\ Y & =\frac{(y_{p}-c_{y})\;Z}{f_{y}},\\ Z & =\frac{z}{S} \end{array}\right.$$

where *S* is the depth scaling factor (typically S=1000 for conversion to meters).

The surface area in the local region is estimated as the average of the areas of two approximating polygons:

(1) $$A=\frac{A_{a}+A_{b}}{2}$$

where

$$A_{a}=\frac{1}{2}\left|\sum_{i=1}^{n}\left(x_{i}\cdot y_{i+1}-x_{i+1}\cdot y_{i}\right)\right|$$

where $A_{a}$ is the area computed using the Gauss formula for a polygon, and $A_{b}$ is the area calculated based on local depth variations:

$$A_{b}=\frac{Z}{f_{x}}\cdot \frac{Z}{f_{y}}$$

The object volume is determined by integrating over the surface area:

$$V=\sum_{(x_{p},y_{p})\in \Omega }A\left(x_{p},y_{p}\right)\cdot \Delta Z\left(x_{p},y_{p}\right),$$

where Ω denotes the object region in the depth map, and $\Delta Z=Z_{bg}-Z_{bk}$ represents the depth difference between the current and background frames.

**Figure 6:**
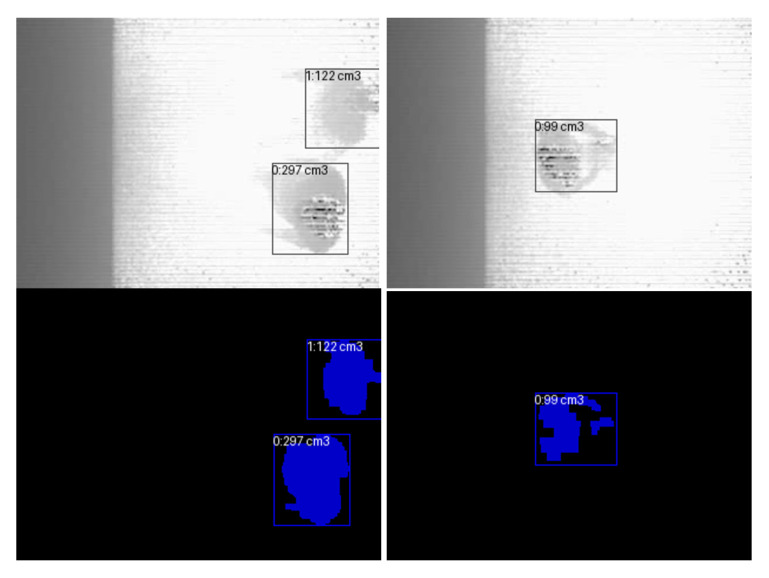
Detecting people based on depth data.

The obtained results are visualized in images where the detected objects are highlighted by bounding boxes reflecting the volumes calculated for them (Figure 6). These frames correspond to the areas where objects were detected when recorded inside a bus, demonstrating how depth-based detection performs in an actual public transport setting, and contain assigned identifiers as well as depth-related metrics.

For subsequent training of the neural network, the coordinates of the bounding boxes are stored in YOLO format. The developed algorithm provides accurate detection of moving objects and assessment of their spatial characteristics in real time, which makes it suitable for the tasks of monitoring passenger flow and analyzing activity in the urban environment.

## 4. Ethical Foundations of Computer Vision Research

### 4.1. Ethical Dimensions of Computer Vision: Principles, Challenges, and Research Perspectives

The rapid development of computer vision technologies has opened up new possibilities for analyzing human behavior, social interactions, and spatial dynamics. These systems enable the collection and interpretation of large amounts of visual data, creating significant potential for research in the fields of sociology, urban studies, and behavioral sciences. However, along with the growing analytical capabilities of such technologies, an equally important aspect arises—an ethical one. The processing of images and video data containing information about people’s personal lives, habits, and individual characteristics is associated with a wide range of risks: violation of confidentiality, unauthorized surveillance, possible discrimination, and vulnerability of personal data.

Moving from a theoretical understanding of risks to the development of practical recommendations, researchers are paying more and more attention to the formation of principles for the responsible use of computer vision technologies. In the study of Golann et al. [26] key ethical principles were formulated, including the protection of personal information and obtaining informed consent, minimizing the amount of data collected, anonymizing information, transparency of research procedures, as well as the implementation of the “Ethics by Design” approach, that is, ethics embedded in the design process. These principles define the directions for creating sustainable and ethically sound computer vision systems in which technology serves not only as an analysis tool, but also as an expression of respect for human dignity and the right to privacy.

Using the example of the New Jersey Families Study [27], a two-week follow-up of 21 families with children aged two to four years (with a total duration of more than 11,470 h of video recordings), the authors showed that working with such data requires not only a high level of technical competence, but also a developed ethical infrastructure. Within the framework of their concept, the principles of confidentiality, transparency, and “ethics by design” are considered as interrelated elements of a unified visual data management system.

Subsequent research has significantly expanded the understanding of ethical aspects in the field of computer vision, going beyond the academic context and covering applied areas. So, in their paper, Sebastian et al. [28] emphasize that using visual analytics technologies to monitor employees involves collecting highly detailed information about human gestures, emotions, and behavior, which makes such systems potentially intrusive. The authors propose an ethical analytical model based on the principles of confidentiality, fairness, transparency and autonomy, which can serve as a basis for responsible design of workplace surveillance systems.

The socio-philosophical aspect of this problem is considered in the work of Waelen [29], where a critical analysis of the political and cultural consequences of the use of computer vision technologies is carried out. The researcher examines these technologies through the prism of power, autonomy and freedom, noting that despite the growing interest in the ethics of artificial intelligence, the field of computer vision remains insufficiently theoretical and needs its own regulatory foundations.

The study by Piasecki and Chen [30] examines in detail the ethical and legal aspects related to compliance with the EU General Data Protection Regulation (GDPR) in the context of smart homes. The authors emphasize the importance of the principle of “data protection by design and by default”, especially in relation to vulnerable groups, that is, children and people in dependent situations. Their work focuses on the need for transparency and accountability in the implementation of computer vision technologies in the daily digital environment. In turn, Stahl et al. [31] have made a significant contribution to the development of this field by applying the Delphi method to analyze the ethical and legal consequences of using artificial intelligence. The results of their research highlight the importance of a systematic, interdisciplinary, and value-based approach to managing risks arising from the introduction of AI and computer vision technologies.

These studies form a holistic scientific field that combines theoretical foundations, normative approaches, and practical mechanisms to ensure ethical responsibility in the field of visual analytics. Table 3 illustrates the main trends in the development of this field and the authors’ contribution to the study of ethical aspects of computer vision applications.

Although the proposed approach demonstrates the potential of ethically oriented computer vision systems, this study has a number of limitations. The current implementation focuses primarily on detecting objects using depth data and does not yet cover a variety of environmental conditions or multimodal sensor inputs. Future work will aim to expand the dataset to include more complex scenarios, for example, scenes with a high density of people and variable lighting, as well as to evaluate the proposed ethical model in various application areas.

Further research should also focus on the development of standardized metrics for assessing ethical compliance in artificial intelligence systems, which will allow for systematic certification and comprehensive analysis of computer vision technologies used in public spaces.

### 4.2. Ethical and Legal Considerations in Passenger Counting Systems

The use of computer vision technologies for passenger counting in public transport is associated with a number of ethical and legal aspects related to the processing of visual data and the protection of personal information. Within the framework of this study, special attention is paid to compliance with the principles of confidentiality and international data protection standards, including the EU General Data Protection Regulation (GDPR).

To reduce the risk of privacy violations, anonymization measures were applied, meaning that the dataset included only images in which passengers’ faces could not be recognized. Object detection was performed based on silhouettes and body poses without the use of biometric features, which fully meets the ethical requirements for visual data processing.

In addition, the proposed system was designed for local (edge) processing of video streams, which eliminates data transmission to external servers and reduces the risk of unauthorized access. Combining YOLOv8 and DeepSORT algorithms with a responsible approach to the use of artificial intelligence has allowed us to achieve a balance between technical accuracy and ethical safety.

The proposed framework confirms that the development of computer vision technologies should be accompanied by a continuous assessment of their impact on human rights, privacy, and social values. In this context, a promising area of future research is the development of universal ethical criteria and auditing standards for visual analysis systems used in the public environment. This approach will not only increase public confidence in intelligent systems but also strengthen the social legitimacy of artificial intelligence technologies.

Although the presented ethical concept sets the framework for responsible use of visual analytics technologies, practical verification of the effectiveness of the proposed system requires a quantitative assessment. It is important to make sure that the implemented algorithms not only comply with regulatory and ethical principles, but also demonstrate reliability when working in real conditions.

The following section describes the experimental setup, the testing methodology, and the results obtained under conditions as close as possible to actual operation.

## 5. Results

### 5.1. Experimental Setup

A series of experiments were conducted using depth-measuring cameras to assess the spatial position and movement of passengers, as well as to improve the accuracy of detecting and tracking objects in conditions of partial overlap and limited visibility. An RGB-D camera with LiDAR technology, Intel RealSense L515, was used as equipment, providing high accuracy of depth measurement in the range from 0.25 to 9 m, and equipped with an integrated inertial module (IMU). This device is optimally suited for three-dimensional tracking tasks [32]. Calibration was carried out according to a standard procedure using temperature compensation [33], which increased the stability of the readings. A hardware trigger with an accuracy of ±1 ms was used to synchronize RGB and depth streams.

### 5.2. Findings

This section presents the key results of the analysis. The study revealed the features of the dataset that affect the accuracy of segmentation and detection of objects, including the distribution of areas, overlap, and differences between group and individual annotations.

The dataset is characterized by a wide range of sizes of marked-up objects, with the bulk being medium-scale objects. This aspect is especially important when choosing the processing scale and designing model architectures for detection and segmentation tasks.

**Figure 7:**
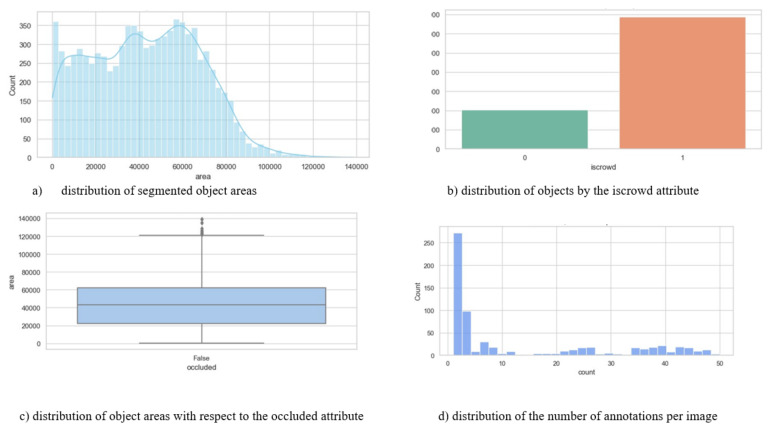
Statistical analysis of the characteristics of segmented objects in the dataset.

Figure 7 shows the main statistical characteristics of the dataset. Subfigure (a) shows the distribution of the areas of objects, which demonstrates a pronounced right-sided asymmetry: most objects are small and medium-sized, while large ones are much less common. Subfigure (b) reflects the relationship between individual and group annotations, where group markup dominates, which is typical for real situations where passengers are located close to each other. Subfigure (c) shows a scale diagram showing the differences in the distribution of the areas of objects depending on the degree of their overlap. Objects without overlaps have more stable area values, while overlapped shapes show greater variability and individual outliers corresponding to large objects. Finally, subfigure (d) illustrates the variation in the number of annotations per image: most scenes contain from 1 to 5 objects, but there are frames with up to 50 annotations, which emphasizes the heterogeneity and high complexity of the dataset under study.

### 5.3. Training the Neural Network Using Open and Proprietary Data

**Figure 8:**
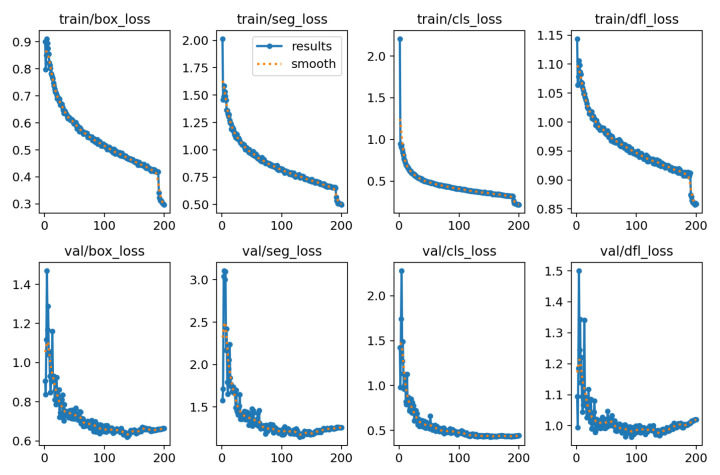
Loss function.

In total 4047 annotated images containing 8918 objects were used to train the segmentation model. 754 images (1596 objects) were selected for testing, and the remaining 3293 images (7322 objects) were used for training. The training was conducted over 200 epochs (Figure 8). Analysis of the learning curve showed that after about the 150th epoch, the model begins to show signs of overfitting.

**Figure 9:**
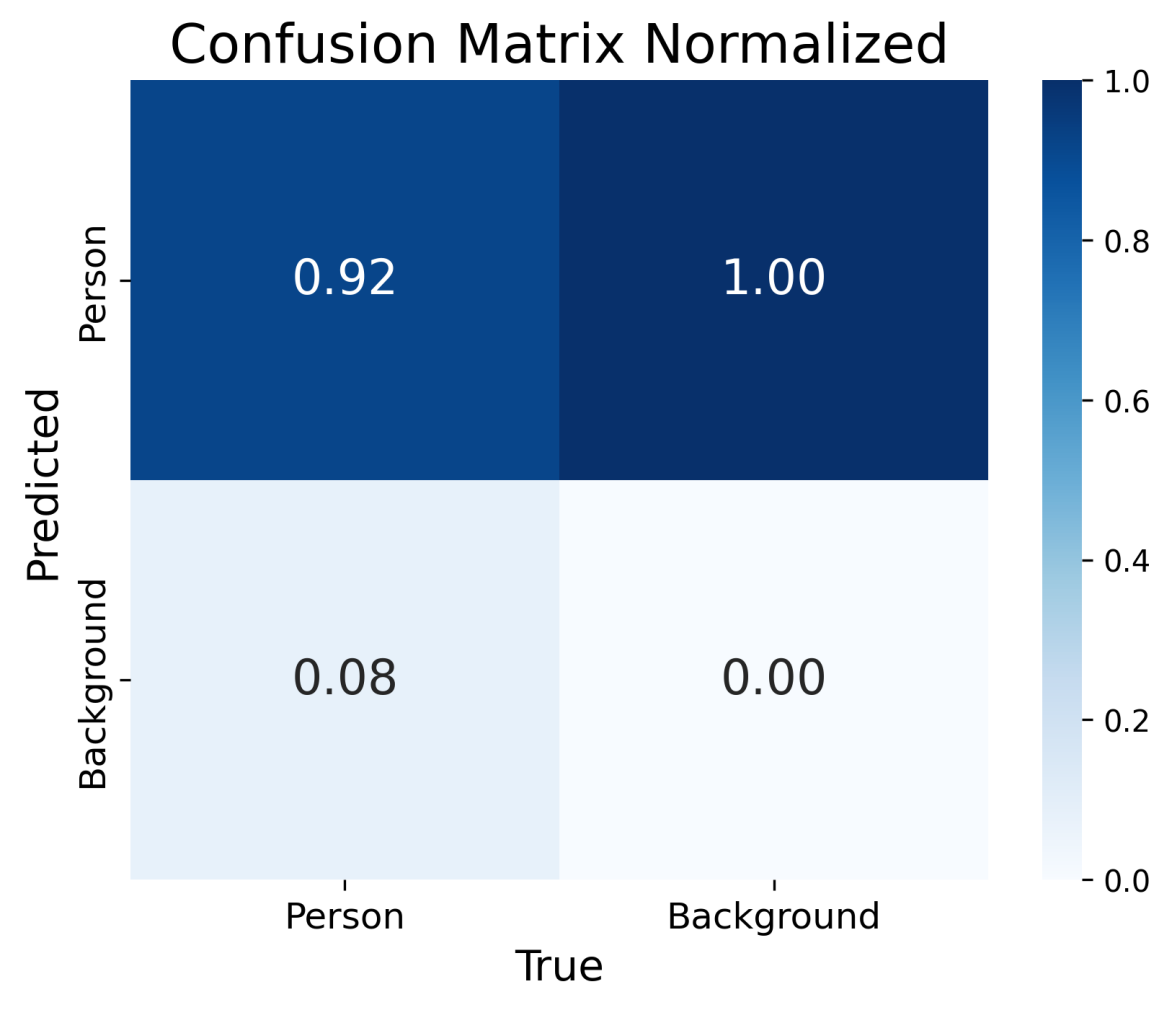
Confusion Matrix.

The confusion matrix demonstrates 92% accuracy of the model (Figure 9).

The normalized error matrix for the YOLOv8 model covers two classes, as “Person” and “Background”. The model correctly classifies objects of the “Person” class in 92% of cases, while 8% are mistakenly assigned to the “Background” class. The “Background” class is recognized with perfect 100% accuracy.

**Figure 10:**
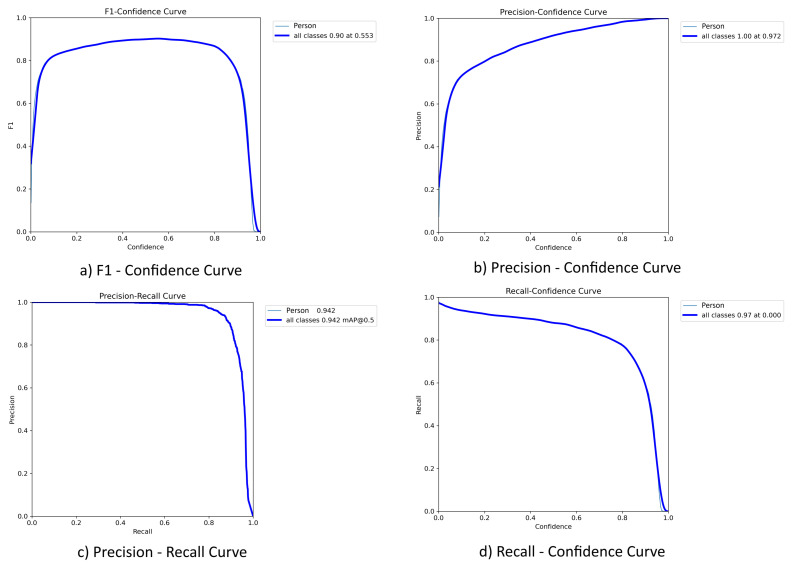
Performance curves of the YOLOv8 model with respect to the confidence threshold.

The curves illustrate the effect of changes in the confidence threshold on the precision, recall, and F1-score indicators, which makes it possible to assess the trade-off between accuracy and completeness when detecting objects of the “Person” class (Figure 10). The YOLOv8 architecture was chosen as the main model due to its high output speed and competitive accuracy, especially with limited computing resources and real-time requirements. The model was initially trained from scratch on open data, and then was retrained on its own dataset. The results of performance metrics are shown in Table 4.

Compared to YOLOv8, the method of He et al. [34] Mask R-CNN implements a two-stage architecture that provides high-precision segmentation of objects at the pixel level. This approach is especially effective in tasks where it is important to accurately distinguish the shape and boundaries of objects. However, using the Region Proposal Network (RPN) mechanism and a separate branch for mask prediction significantly increases the computational complexity of the model. As a result, Mask R-CNN requires more resources and processing time, which limits its use in real-time systems and on devices with limited hardware capabilities.

The DETR model [35] is based on the transformer architecture and implements an end-to-end approach to object detection. Compared to classical methods, it eliminates the need to use heuristic components, such as anchor frames or non-maximum suppression (NMS). Instead, the model directly correlates predictions with true labels using the Hungarian algorithm, which ensures high accuracy and elegant architecture. However, due to the complexity of training, DETR requires considerable time to set up and demonstrates a relatively low processing speed, which limits its use in real-time tasks.

Considering these features, the YOLOv8 model was chosen to solve the detection problem, which demonstrated the most successful balance between accuracy and computational efficiency. Due to this, it proved to be the optimal choice for systems with limited hardware resources and strict performance requirements. Object detection was performed using YOLOv8, pre-trained on the COCO dataset.

A modified version of the DeepSORT algorithm [13] was used to track objects, supplemented by the use of deep data. This made it possible to increase the stability of tracking and ensure stable tracking of objects even in dynamic scenes. To further improve the quality, an adaptive bilinear filter [36] was used, which effectively suppressed noise and helped maintain clear boundaries of objects.

The results of comparative testing of various algorithms on the selected data set are shown in Table 5. They show differences in accuracy and performance of solutions, confirming the effectiveness of the proposed model configuration.

An algorithmic architecture has been developed for counting objects based on the analysis of trajectories crossing a virtual boundary. The proposed method reliably determines the direction of movement (entrance/exit) and performs spatiotemporal analysis with high accuracy and noise resistance. The algorithm generates intersection statistics, object identifiers, and vectors of their movement directions. Due to the use of polynomial smoothing, the method is resistant to short-term tracking errors, but its accuracy depends on the stability of the identifiers and the reliability of the tracking system. In addition, the use of computer vision systems in public spaces affects important ethical and legal aspects, which are discussed in detail in the next section.

**Figure 11:**
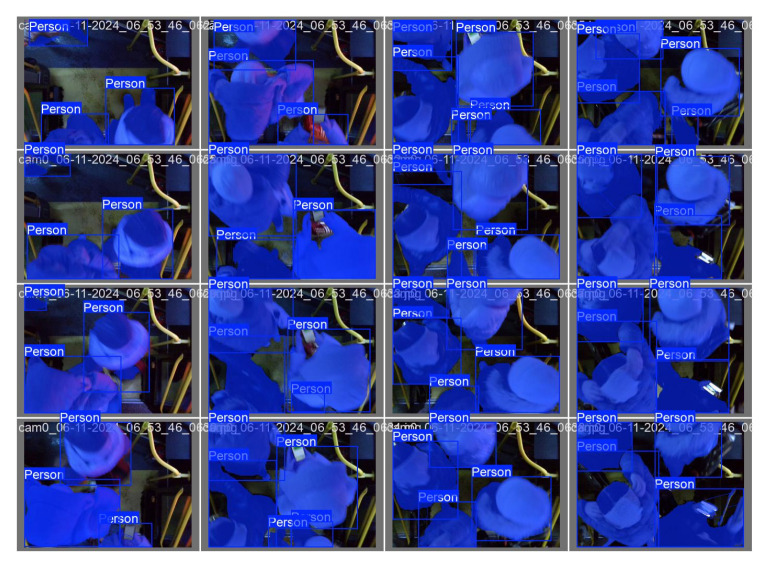
Visualization of YOLOv8 predictions for the “Person” class on test images.

Figure 11 presents examples of the results obtained when testing the YOLOv8 model. The algorithm correctly defines objects of the “Person” class, forming both the boundaries of the bounding rectangles and segmentation masks. It can be noted that the model retains high accuracy even with partial overlaps, changes in illumination and various orientations of objects. The results obtained confirm the stability and generalization ability of the model, which is especially important when analyzing complex visual scenes.

**Figure 12:**
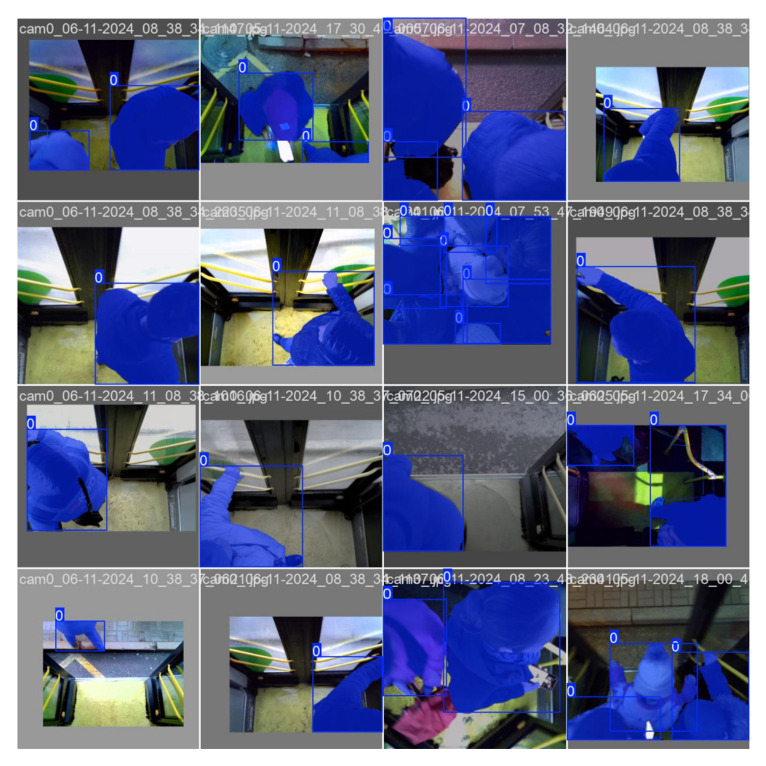
Examples of YOLOv8 performance in real-world scenarios (public transport interior).

Figure 12 shows examples of how the YOLOv8 model works on video recordings obtained under real-world operating conditions, including inside a public transport cabin. The images clearly show that the model is able to confidently recognize and segment objects of the “Person” class, even when there is a large crowd of people in the frame, there are overlapping figures or changes in posture and lighting. Despite the dynamics of the scene and the limited view, the YOLOv8 retains high contour detection accuracy and resistance to visual noise [37].

**Figure 13:**
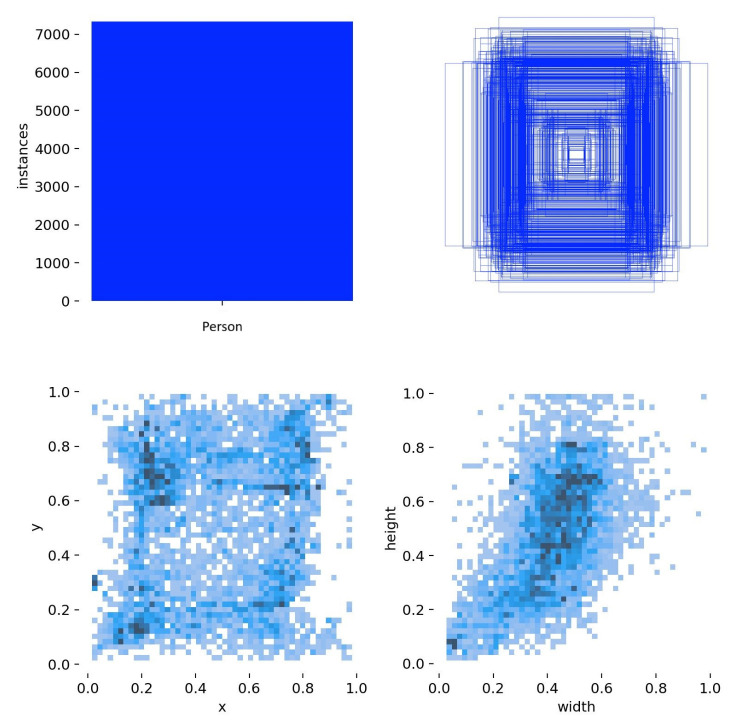
Spatial distribution of annotated objects of the “Person” class.

Figure 13 summarizes the spatial and geometric characteristics of all annotated “Person” bounding boxes in the dataset used in the training of the model.

The upper-left panel shows a histogram of the distribution of objects (about 7000 annotations), which clearly shows the imbalance of classes in the sample. The upper right panel shows the overlap of all the bounding boxes, which allows to estimate the spatial distribution of objects in the frame, most of them are concentrated in the central region of the image. The lower-left panel shows the distribution of coordinates of the object centers, reflecting the areas with the highest density, which is related to the peculiarities of shooting conditions. Finally, the lower right panel illustrates the size distribution of the bounding boxes, where the predominance of small and medium-sized objects is clearly visible. It is important to take this fact into account when configuring the model parameters and choosing the data processing scale.

### 5.4. Comparison with the State-of-the-Art Methods

Accurate and timely assessment of the number of people (occupation counting) plays a key role in ensuring safe and efficient operation of buildings and transport infrastructure. Such systems help to manage demand, improve service quality, and reduce energy consumption by optimizing the load on ventilation and climate control systems (HVAC).

Based on the analysis of existing scientific sources, various approaches to passenger counting and assessment of their accuracy were considered. The main metrics used in these studies, such as Accuracy, MAE, RMSE, and X-Accuracy, were compared and unified to perform the comparative analysis presented in Table 6. The proposed table comparatively presents field results across diverse application contexts (on-bus/off-bus, metro, in-carriage episodes, and multi-camera indoor systems), providing a methodological basis for subsequent experimental evaluation and for designing occupant-centric control (OCC) scenarios.

**Figure 14:**
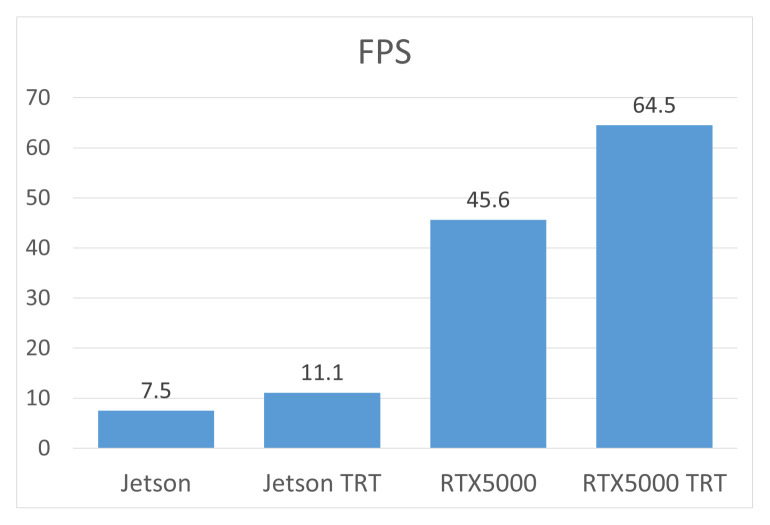
Comparative performance (FPS) of the algorithm across different devices.

To evaluate the performance of the algorithm, a series of tests was conducted on two hardware platforms—Jetson Nano and NVIDIA RTX5000. The tests were carried out both with and without TensorRT optimization, which allowed to determine the impact of faster calculations on the overall performance of the model. The results shown in Figure 14 demonstrate that using TensorRT can significantly reduce data processing time, especially when performing calculations on graphics processing units (GPUs). This approach ensures more efficient use of computing resources and improves the overall performance of the model. After analyzing the results obtained, the system underwent additional experimental testing under real-world operating conditions. This allowed us to confirm its reliability, stability of operation, and suitability for practical use in scenarios close to the real environment.

### 5.5. Experimental Results and Discussion

**Figure 15:**
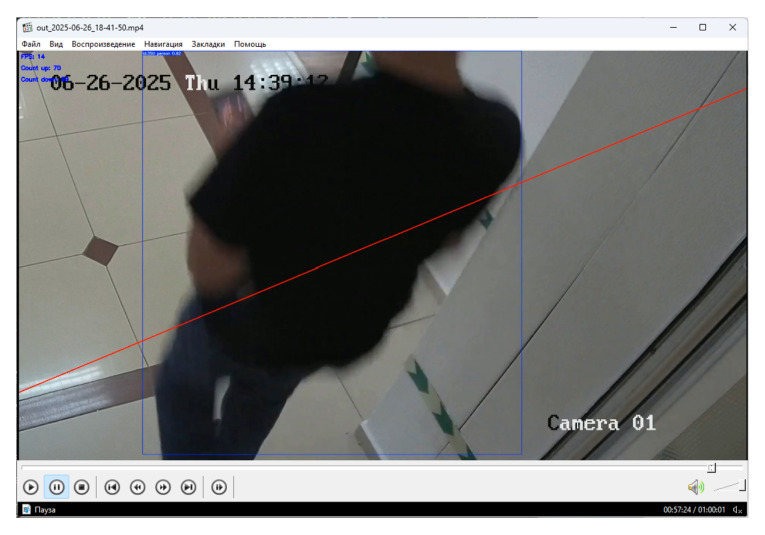
Frame from the test video used for evaluating the people-counting algorithm.

To evaluate the effectiveness of the algorithm for counting people, a three-hour video recorded in advance (Figure 15) was used, which included various scenarios of human movement.

**Figure 16:**
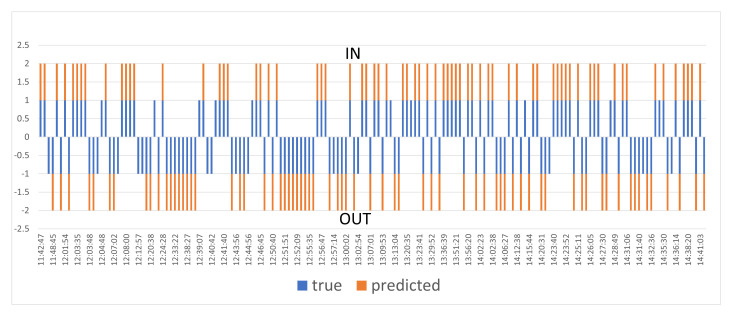
Comparison of actual and predicted number of people over the time scale.

Figure 16 presents the dynamics of people’s entrances and exits during the entire observation period. The blue columns reflect the manual calculation data, and the orange columns reflect the results obtained by the algorithm. There is a strong correlation between the actual and predicted values, which confirms the reliability and accuracy of the developed system, despite minor discrepancies in certain time intervals.

**Figure 17:**
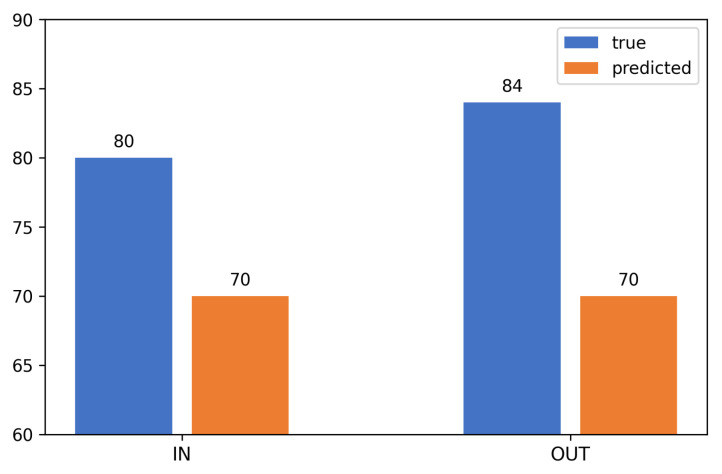
Overall accuracy of the people-counting algorithm: 140 correct cases out of 164 (85 percent).

The overall accuracy of the algorithm was 85%. Of the 80 actual inputs, the system correctly identified 70, and of the 84 outputs, it also identified 70. Thus, 140 of the 164 recorded events were accurately recognized, which is clearly shown in Figure 17.

The developed YOLOv8-based passenger counting algorithm has shown stable and high efficiency (85%) in processing video streams with various types of traffic. Experimental tests have confirmed the reliable correspondence of the model’s predictions to real values, and also revealed a significant acceleration of calculations using TensorRT optimization.

A manually annotated set of 2000 images served as a qualitative empirical basis for further improving the accuracy of the model and adapting it to various application scenarios. The experimental results obtained form the basis for the final analysis and determination of future research directions.

## 6. Conclusions

The paper presents a comprehensive analysis and practical implementation of an automatic passenger detection and counting system in urban transport using modern computer vision algorithms. Analysis of existing methods has shown significant differences in accuracy and processing speed, which highlights the need to create models that ensure high accuracy when working in real time. An important contribution of this research was the creation of our own dataset of 4047 images and 8918 annotated objects, covering a wide range of scenes and shooting conditions to increase the stability and generalizing ability of models. As part of the study, the YOLOv8 model was adapted and tested for detecting and segmenting objects of the “Person” class, and the use of RGB, stereo, and depth cameras increased the algorithm’s resistance to background changes and partial overlaps. The results showed high accuracy (mAP@50 = 0.95, F1 = 0.90) and reliable operation of the model in urban transport conditions. Optimization via TensorRT has improved performance without loss of accuracy, ensuring real-time operation. Compared to Mask R-CNN and DETR, the YOLOv8 model demonstrated the best balance between recognition speed and quality with limited computing resources. In addition to the technical aspects, the study addresses issues of ethics and confidentiality. To comply with the principles of anonymity, the possibility of identifying individuals is excluded, and data processing is performed locally without transmission to external servers, which complies with GDPR requirements. The results confirm the potential of computer vision systems as a reliable and scalable alternative to traditional sensory passenger counting methods. In the future, it is planned to expand the dataset through nighttime and crowded scenes, introduce multi-camera integration to prevent repeated counting, and optimize the model for embedded devices, which will increase the versatility and energy efficiency of the technology within the framework of the smart city concept.

## Figures and Tables

**Table 1:** Comparative table of passenger counting methods.

Paper	Datasets	mAP/Acc	FPS
Ren et al. [2]	Custom	64% mAP	>40
Sun et al. [10]	PCDS	92% acc	45
Kusuma et al. [11]	OI Dataset	73% mAP	n/a
Baumann et al. [3]	PCDS	97% mAP	∼3.2
Radovan et al. [17]	Various	n/a	n/a

**Table 2:** Example of a dataframe corresponding to the figure.

ID	Image ID	Category ID	Area	Iscrowd	Iscrowd	Width	Height
0	1	1	12,877.0	1	False	640	480
1	2	1	27,817.0	0	False	640	480
2	3	1	53,658.0	0	False	640	480
3	4	1	9483.0	0	False	640	480
4	5	1	10,115.0	1	False	640	480

**Table 3:** Key studies addressing ethical aspects of computer vision applications.

No.	Authors	Year	Research Focus	Key Ethical Principles
1	Golann et al. [26]	2024	Ethical and methodological challenges in working with video data (New Jersey Families Study)	Privacy, informed consent, anonymization, transparency
2	Sebastian et al. [28]	2025	Ethical framework for AI-based workplace monitoring	Privacy, fairness, transparency, autonomy
3	Waelen et al. [29]	2024	Critical review of socio-political dimensions of computer vision	Power, autonomy, social justice, freedom
4	Piasecki et al. [30]	2022	Ethical and legal aspects of smart homes and GDPR compliance	Data protection, transparency, accountability
5	Stahl et al. [31]	2023	Delphi study on ethical and human rights implications of AI	Responsibility, systemic approach, interdisciplinarity

**Table 4:** Performance metrics approaches.

Metric	Bounding Box (B)	Mask (M)
Precision	0.91971	0.93538
Recall	0.86848	0.8526
mAP@50	0.93335	0.93295
mAP@50-95	0.74044	0.67564

**Table 5:** Example of a dataframe corresponding to the figure.

Method	MOTA	IDF1	Depth	Latency	Memory Consumption
SORT [12]	0.58	0.70	No	10 ms	500 Mb
DeepSORT [13]	0.65	0.78	Partially	45 ms	1.2 Gb
FairMOT [14]	0.67	0.80	No	35 ms	1.5 Gb

**Table 6:** Field results for people/occupancy counting in public transport and buildings.

Location of People	Ground Truth	Estimated	Accuracy
On the bus [18]	3126	3406	91.8%
Outside the bus [18]	10,792	10,428	96.6%
metro train [38]	low-ridership	-	83.53% and 94.87%
Video clips [39]	145 landings	-	83.53% and 94.87%
Video clips [39]	177 boarding	-	83.53% and 94.87%

## Data Availability

The data presented in this study are available on request from the corresponding author.

## Abbreviations

mAP	Mean Average Precision
FPS	Frames Per Second
R-CNN	Region-based Convolutional Neural Network
DETR	Detection Transformer
ROI	Region of Interest
MOTA	Multiple Object Tracking Accuracy
IDF1	ID-based F1 Score
RGB-D	Red–Green–Blue plus Depth
COCO	Common Objects in Context Dataset
PCDS	People Counting Dataset
GDPR	General Data Protection Regulation
RPN	Region Proposal Network
NMS	Non-Maximum Suppression
CBAM	Convolutional Block Attention Module
EIOU	Enhanced Intersection over Union
LiDAR	Light Detection and Ranging
IMU	Inertial Measurement Unit
